# Individualized immunotherapy for immune reconstitution-associated cortical encephalitis in an HIV-positive cryptococcal meningitis patient: a case report

**DOI:** 10.3389/fimmu.2026.1805701

**Published:** 2026-03-30

**Authors:** Wen Wang, Shi Tang, Jun Yang, Ying Wen

**Affiliations:** 1 Department of Infectious Diseases, The First Affiliated Hospital of China Medical University, Shenyang, Liaoning, China; 2 Neurology Department, The First Affiliated Hospital of China Medical University, Shenyang, Liaoning, China

**Keywords:** cortical encephalitis, cryptococcal meningitis, HIV, immune reconstitution inflammatory syndrome (IRIS), mycophenolate mofetil (MMF)

## Abstract

**Background:**

Neurological immune reconstitution inflammatory syndrome (IRIS) in HIV can present variably. We report a case of steroid-dependent, recurrent cortical encephalitis with a distinctive migratory Magnetic Resonance Imaging (MRI) pattern following cryptococcal meningitis.

**Case presentation:**

A 38-year-old male with advanced HIV (CD4 11/μL) developed cryptococcal meningitis. After antifungal induction and switching to effective antiretroviral therapy (bictegravir/emtricitabine/tenofovir alafenamide), he suffered recurrent neurological episodes. Serial brain MRIs showed sequentially appearing and resolving T2/Fluid-Attenuated Inversion Recovery (FLAIR) hyperintensities in the bilateral frontal lobes, left cingulate gyrus, and left occipital cortex, despite negative infectious and autoimmune workup. Symptoms were steroid-responsive but relapsed upon tapering. Lasting remission was achieved only after adding mycophenolate mofetil (MMF), allowing corticosteroid withdrawal.

**Conclusions:**

This case describes a “migratory cortical encephalitis” phenotype of Central Nervous System (CNS)-IRIS. It highlights that cortical grey matter can be the primary target in severe IRIS and illustrates the utility of steroid-sparing agents like MMF for managing refractory, steroid-dependent neuroinflammation in this context.

## Introduction

Cryptococcal meningitis (CM) remains a significant cause of morbidity and mortality in persons with advanced HIV infection, particularly those with CD4 counts below 100 cells/μL (1). The introduction of combination antiretroviral therapy (ART) can precipitate immune reconstitution inflammatory syndrome (IRIS), a paradoxical clinical worsening due to an exuberant immune response against latent or treated opportunistic pathogens (2). Neurological CM-IRIS presents a severe management challenge, with an incidence ranging from 6% to 45% (3), typically manifesting as recurrent meningitis symptoms, new or enlarging cryptococcomas, or increased intracranial pressure weeks to months after ART initiation (3).

The radiological spectrum of CM-IRIS often includes enhancing parenchymal lesions or pronounced meningeal enhancement, differentiating it from the pseudocysts and infarcts more common in untreated CM (4, 5). Management typically involves corticosteroids, but refractory cases pose a significant therapeutic dilemma (4, 6).

We herein present a case of advanced HIV complicated by ART failure with acquired resistance, subsequent CM, and a complex, steroid-dependent course of recurrent Central Nervous System (CNS)-IRIS manifesting as migratory cortical encephalitis. This report aims to discuss the management of refractory IRIS and underscore vital preventive strategies.

## Case presentation

A 38-year-old man was diagnosed with HIV-1 infection in January 2022. Baseline evaluation revealed a CD4 T-cell count of 11 cells/μL, an HIV viral load of 1.72 x 10^5^ IU/mL, and no primary antiretroviral resistance mutations. He commenced ART with lamivudine, tenofovir disoproxil fumarate, and efavirenz after receiving standard adherence counseling and with reported excellent adherence. However, due to personal reasons, he did not attend regular follow-up visits, and no interim CD4 count or HIV viral load monitoring was performed between diagnosis in January 2022 and presentation in September 2022.

In September 2022, he presented with a 5-day history of severe headache, nausea, vomiting, and intermittent generalized tonic-clonic seizures. Neurological examination revealed lethargy without focal deficits. His CD4 count was 7 cells/μL, and HIV RNA was 2.19 x 10^4^ IU/mL. Genotypic resistance testing revealed nucleoside reverse transcriptase inhibitors (NRTIs)-associated mutations (Y115F, M184V) and nonnucleoside reverse transcriptase inhibitors (NNRTIs)-associated mutations (V179D, G190S), confirming high-level resistance to lamivudine and efavirenz. Lumbar puncture demonstrated an opening pressure of 280 mmH_2_O. Cerebrospinal fluid (CSF) analysis showed a mild lymphocytic pleocytosis (15 cells/μL) and elevated protein (650 mg/L). India ink stain was positive for encapsulated yeasts, and culture was confirmed as *Cryptococcus neoformans.* Brain MRI was unremarkable.

A diagnosis of CM with ART failure was made. Induction antifungal therapy was started with amphotericin B cholesteryl sulfate complex (300 mg/day IV) plus fluconazole (800 mg/day IV) and flucytosine (1.5g orally every 6 hours). ART was switched to bictegravir/emtricitabine/tenofovir alafenamide (B/F/TAF) two weeks later. Following a 3-week induction, consolidation therapy with fluconazole and flucytosine continued for 8 weeks. By December 2022, his symptoms had resolved. Repeat lumbar puncture showed negative CSF culture, though cryptococcal capsular antigen (CrAg) [Beijing Hightrust Diagnostics Laboratory] was detectable at 16.17 ng/mL. His CD4 count had risen to 69 cells/μL.

**Figure 1:**
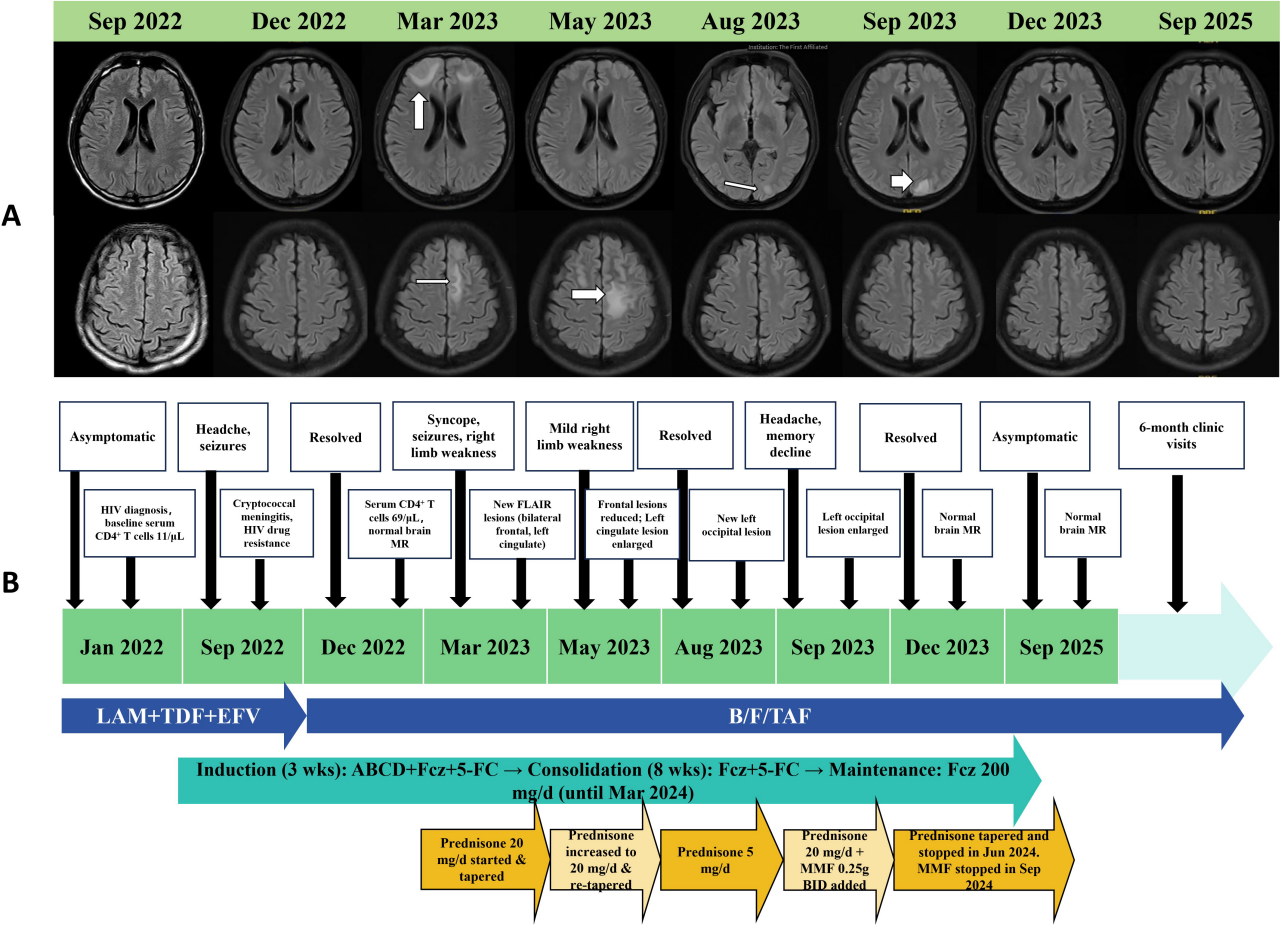
Brain MRI findings, the timeline for treatment adjustment and follow-up of the patient with HIV-associated cryptococcal meningitis and recurrent CNS immune reconstitution inflammatory syndrome (IRIS). **(A)** Serial axial FLAIR/T2-weighted images at different time points during the clinical course. Sep 2022: At the initial diagnosis of cryptococcal meningitis. The brain MRI scan is unremarkable. Dec 2022: Following antifungal induction therapy. The MRI remains within normal limits. Mar 2023: During the first episode of suspected CNS-IRIS. Images show new, patchy, cortical-predominant hyperintense lesions in the bilateral frontal lobes and left cingulate gyrus (white arrows). May 2023: Follow-up after initial corticosteroid therapy. The frontal lobe lesions have decreased in size, while the lesion in the left cingulate gyrus appears enlarged (white arrows). Aug 2023: After corticosteroid taper. The frontal and cingulate lesions have resolved. A new, focal hyperintense lesion is present in the left occipital cortex (white arrow). Sep 2023: Upon recurrence of neurological symptoms. The left occipital lobe lesion shows significant enlargement (white arrow). Dec 2023: Following combination therapy with corticosteroids and mycophenolate mofetil (MMF). The previously seen left occipital lesion has completely resolved. Sep 2025: At the final long-term follow-up. The brain MRI is normal, with no evidence of residual or new lesions. Abnormalities are indicated by white arrows. **(B)** Schematic timeline illustrating the key clinical events, imaging findings, and corresponding adjustments in antifungal, antiretroviral, and immunomodulatory therapies over the course of the patient’s illness. Abbreviations: LAM, lamivudine; TDF, tenofovir disoproxil fumarate; EFV, efavirenz; B/F/TAF, bictegravir/emtricitabine/tenofovir alafenamide; ABCD, amphotericin B cholesteryl sulfate complex; Fcz, fluconazole; 5-FC, flucytosine; MMF, mycophenolate mofetil; BID, twice daily.

In March 2023, he presented acutely with right limb weakness, syncope, and seizures. At this time, his CD4 count was 107 cells/μL. CSF analysis revealed elevated pressure (247.5 mmH_2_O), lymphocytic pleocytosis (21 cells/μL), and markedly elevated protein (1005 mg/L). CSF CrAg was 3.82 ng/mL; culture and metagenomic next-generation sequencing (mNGS) were negative. Blood and CSF HIV RNA were undetectable. Autoimmune encephalitis antibody panels [Nanjing Simcere Diagnostics Laboratory] were negative. Brain MRI showed new, patchy, cortical-predominant T2/FLAIR hyperintensities in the bilateral frontal lobes and left cingulate gyrus, with subtle leptomeningeal enhancement (**Figure 1**). A diagnosis of CNS-IRIS was made. He responded well to prednisone 20 mg daily, tapered over 8 weeks to 5 mg daily.

In May 2023, a follow-up MRI revealed reduction in the bilateral frontal lesions but expansion of the left cingulate gyrus lesion. Prednisone was increased to 20 mg daily and slowly tapered over 12 weeks. By August 2023, the frontal lesions had resolved, but a new lesion appeared in the left occipital cortex. He was asymptomatic on 5 mg prednisone. In September 2023, headache and memory decline recurred. MRI confirmed enlargement of the occipital lesion. Therapy with prednisone 20 mg daily and mycophenolate mofetil (MMF) 500 mg daily was initiated, leading to symptom resolution. By December 2023, the occipital lesion had completely resolved. Maintenance fluconazole was stopped in March 2024. Prednisone was gradually tapered and discontinued by June 2024 while continuing MMF. In September 2024, CSF CrAg was negative, and MMF was discontinued. At the last follow-up in September 2025, he remained asymptomatic with a normal neurological exam, normal brain MRI, and a CD4 count of 411 cells/μL. A timeline of key events and treatment is summarized in **Table 1**. The changes in this patient’s brain MR scans are shown in **Figure 1A**. The timeline for treatment adjustment and follow-up of this patient is clearly outlined in **Figure 1B**.

**Table 1:** Summary of clinical course, investigations, and treatment from diagnosis to follow-up in a patient with HIV-associated cryptococcal meningitis and CNS-immune reconstitution inflammatory syndrome (IRIS).

Date of Examination and Treatment	Reference range	Baseline (Jan 2022)	Sep 2022	Dec 2022	Mar 2023	Sep 2023	Dec 2023	Jun 2024	Sep 2024	Sep 2025
Event		no symptoms	headache, convulsion	relief	syncope, convulsion	headache, memory decline	relief	no symptoms	no symptoms	no symptoms
CSF cell count (cells/μL)	0-8		27	4	21	11	NA	NA	3	9
CSF protein (mg/L)	120-600		1120	534	1005	1002	NA	NA	508	573
Lumbar puncture pressure(mmH_2_O)	80-180		280	202.5	247.5	248	NA	NA	248	175
CSF smear for Cryptococcus	–		+	–	–	–	NA	NA	–	–
CSF cryptococcus capsular antigen			NA	16.17	3.82	0.38	NA	NA	<0.05	<0.05
Serum cryptococcus capsular antigen			NA	NA	NA	NA	NA	9.98	6.33	NA
plasma CD4^+^ T cell count (cells/μL)	410-1590	11	7	69	107	145	184	215	226	239
plasma CD8^+^ T cell count (cells/μL)	190-1140	513	542	1366	923	1006	1229	981	881	1191
plasma HIV-RNA (copies/mL)	<20	1.72× 10^5^	2.192× 10^4^		<20					<20
anticryptococcal regimen			ABCD+Fcz+5-FC	Fcz 200mg/d	Fcz 200mg/d	Fcz 200mg/d	Fcz withdrawn in March 2024			
ART regimen		LAM/TDF/EFV	B/F/TAF	B/F/TAF	B/F/TAF	B/F/TAF	B/F/TAF	B/F/TAF	B/F/TAF	B/F/TAF
Corticosteroid therapy					prednisone20mg/d	Prednisone 20mg/d+MMF 0.25g BID	Prednisone 15mg/d+ MMF	Prednisone stopped, continue MMF	MMF stopped	

CSF, cerebrospinal fluid; ABCD, amphotericin B cholesteryl sulfate complex; Fcz, fluconazole; 5-FC, flucytosine; ART, antiretroviral therapy; LAM, lamivudine; TDF, tenofovir disoproxil fumarate; EFV, efavirenz; B/F/TAF, bictegravir sodium, emtricitabine and tenofovir alafenamide fumarate tablets; MMF, mycophenolate mofetil.

## Discussion

This case illustrates the multifaceted challenges in managing advanced HIV infection, encompassing initial ART failure with acquired resistance, subsequent CM, and a complex, steroid-dependent CNS-IRIS following ART optimization. The clinical course delineates two distinct phases: opportunistic infection during profound immunosuppression, followed by an inflammatory neurological disorder coinciding with immune recovery. The differential diagnosis centered on excluding cryptococcal relapse, other opportunistic CNS infections, or HIV-related neuropathology. The persistent negativity of CSF cryptococcal culture with decreasing CrAg titers, undetectable HIV RNA, and negative comprehensive microbiological and autoimmune panels collectively pointed towards an exaggerated inflammatory response to residual antigen—IRIS—rather than active infection (7).

Regarding the timing of ART, initiating effective therapy 2 weeks into CM induction, as in this case, may increase IRIS risk compared to the recommended 4–6 weeks after antifungal therapy starts. The neuroradiological manifestations here were distinctive. Our patient exhibited a “migratory cortical encephalitis” pattern, with lesions sequentially appearing in the bilateral frontal lobes, left cingulate gyrus, and left occipital cortex. This predominantly cortical involvement with migratory, reversible lesions more closely resembles certain autoimmune encephalitides than the white matter-predominant pattern of HIV-associated CD8 encephalitis (8), broadening the recognized spectrum of CNS-IRIS. The precise mechanisms underlying this cortical predilection remain speculative but may involve antigen persistence within perivascular spaces or meninges, leading to compartmentalized inflammation. Cortical grey matter contains rich microglial networks and perivascular macrophages that can serve as antigen-presenting cells, potentially attracting activated CD8+ T lymphocytes upon immune recovery. This pattern shares features with HIV-associated CD8+ T-cell encephalitis, though the latter typically shows white matter predominance (8). Recent literature suggests that persistent cryptococcal antigen may trigger a dysregulated T-cell response targeting cortical structures, possibly explaining the migratory cortical lesions observed in our patient (3, 5).

The dynamic detection of CrAg provided a critical interpretive lens. Detectable serum and low-level CSF CrAg persisted well beyond microbiological cure. In the context of immune reconstitution, this likely represents ongoing antigen clearance and immunologic stimulus driving IRIS flares, rather than indicating therapeutic failure (9, 10). It is important to recognize that non-viable cryptococcal structures may persist in the CSF for many months after successful antifungal therapy; their slow clearance carries no clinical significance, and culture negativity remains the decisive parameter for cure (10). Monitoring CSF CrAg trajectory may aid in gauging IRIS activity, but should not supersede culture results in assessing treatment response.

The management of this steroid-dependent IRIS necessitated advanced immunomodulation. While initial prednisone was effective, relapses occurred upon tapering below a threshold dose. The introduction of the steroid-sparing agent MMF ultimately achieved sustained remission and allowed for safe corticosteroid withdrawal, aligning with guideline suggestions for severe cases (11, 12).

This case has three principal limitations. First, baseline serum CrAg screening, strongly recommended for ART-naïve individuals with CD4 <100 cells/μL (11, 13), was not performed and could have potentially averted CM. Second, virological monitoring was inadequate; earlier detection of virological failure (e.g., at 4–8 weeks post-ART) might have prompted a timely ART switch and possibly prevented the subsequent CM and IRIS cascade (14). Third, immunological monitoring was also suboptimal. The persistently low CD4 counts that failed to rise substantially above 100 cells/μL should have prompted consideration not only of possible virological failure but also of occult cryptococcal antigenemia. Even in patients already established on ART, inadequate immune recovery warrants CrAg screening, as cryptococcal disease can occur or progress despite ART. Earlier recognition of poor immune reconstitution might have offered an opportunity to detect subclinical cryptococcal infection before the onset of fulminant meningitis and the subsequent severe IRIS cascade.

## Conclusion

This report details a recurrent, steroid-dependent CM-IRIS manifesting as migratory cortical encephalitis. The unique cortical-predominant imaging presentation expands the clinico-radiological spectrum of IRIS. Dynamic CrAg monitoring can guide clinical assessment. For refractory IRIS, adjunctive steroid-sparing agents like MMF represent a viable management strategy. This case reinforces the paramount importance of rigorous virological monitoring early in ART and pre-ART cryptococcal screening in persons with advanced HIV to prevent severe complications.

## Data Availability

The original contributions presented in the study are included in the article/**Supplementary Material**. Further inquiries can be directed to the corresponding author.
